# microRNA involvement in the onset and progression of Barrett's esophagus: a systematic review

**DOI:** 10.18632/oncotarget.24145

**Published:** 2018-01-11

**Authors:** Reilly J. Clark, Michael P. Craig, Sangeeta Agrawal, Madhavi Kadakia

**Affiliations:** ^1^ Department of Biochemistry and Molecular Biology, Wright State University, Dayton, OH, USA; ^2^ Dayton VA Medical Center, Dayton, OH, USA

**Keywords:** Barrett’s esophagus, microRNA, EAC, biomarker

## Abstract

Esophageal adenocarcinoma (EAC) is a highly aggressive malignancy that develops from Barrett's esophagus (BE), an intestinal metaplasia of the distal esophagus. microRNAs (miRNAs), short non-coding regulatory RNAs, are frequently dysregulated in BE and are thought to play key roles in the onset of BE and its progression to EAC. miRNAs thus have potential diagnostic and prognostic value and are increasingly being used as cancer biomarkers. This review summarizes the current literature related to miRNAs that are dysregulated in BE within the context of Hedgehog, Notch, MAPK, NF kappa-B, Wnt and epithelial-mesenchymal transition (EMT) signaling which are thought to drive BE onset and progression. This comprehensive analysis of miRNAs and their associated signaling in the regulation of BE provides an overview of vital discoveries in this field and highlights gaps in our understanding of BE pathophysiology that warrant further investigation.

## INTRODUCTION

MicroRNAs (miRNAs) are evolutionarily conserved small non-coding RNAs of approximately 18-25 nucleotides in length which regulate gene expression by binding to the 3’-untranslated region (3’-UTR) of target mRNAs to inhibit their translation or facilitate their degradation [1]. miRNAs have been shown to regulate cell growth, differentiation and migration and are frequently dysregulated in cancer. Accordingly, there is considerable interest in identifying miRNA signatures characteristic of disease stage or therapeutic responsiveness. Further, microRNAs are being increasingly used clinically as cancer biomarkers because they frequently exhibit tissue-specific differences in expression, are stable in formalin-fixed tissues and blood and may be profiled without the need for an invasive muscle biopsy [2, 3]. Serum miRNA biomarkers have particular appeal in diseases like Esophageal Adenocarcinoma (EAC) which are associated with extended monitoring periods, poor early detection, and costly or invasive methodologies.

Esophageal adenocarcinoma (EAC) is the dominant histologic type of esophageal cancer in Western society and its incidence is increasing. The precursor lesion for EAC, termed Barrett's esophagus (BE), is a metaplastic lesion of the distal esophagus in which the normal esophageal squamous epithelium is replaced by columnar epithelia and goblet cells. Patients with BE are 30–40 times more likely to develop EAC [4]. Gastroesophageal reflux disease (GERD) is the only known risk factor for BE, and its prevalence is estimated to be as high as 30% in the United States [5]. Currently, white light endoscopy (WLE) with biopsy is the standard for diagnosis of BE and EAC [6]. The mortality rate in BE patients who develop EAC drops by 61% when patients are regularly monitored by WLE [7]. However, BE lesions that occur at the squamocolumnar junction or in the esophageal submucosal glands are not easily identifiable through WLE and thus not biopsied [8]. In addition, inflammation in the reflux-damaged epithelium can be misdiagnosed as dysplasia by WLE with biopsy [9]. Patient anxiety, adverse patient responses to sedatives, and high procedural costs also undermine the broad utility of this approach. Since the incidence of EAC in BE patients is only 0.55%, the cost-effectiveness of surveillance monitoring by WLE and biopsy has also been questioned by recent meta-analyses [7, 10]. It is therefore necessary to identify BE patients most at risk for EAC, allowing BE patients with negligible risk to forego the cost, discomfort, and risks of repeated endoscopic procedures. Accordingly, there is a need for noninvasive serum biomarkers to identify the minority of patients at high risk for dysplasia who will benefit from surveillance.

BE and EAC research has identified a number of potential effectors of BE and EAC development which may have potential diagnostic or prognostic value. Proteins identified as differentially regulated in BE and EAC patients include Trefoil Factor 3, Anterior gradient 2, p16, villin, MUC2, and various columnar epithelial cytokeratins [11–15]. In addition, the differential expression of p63 in BE lesions has suggested a connection to normal mammalian embryonic development [16, 17]. However, none of these proteins or any combination thereof are in clinical use as biomarkers for BE or EAC. Fortunately, a number of miRNAs have been proposed as potential biomarkers for the diagnosis and monitoring of BE [18–26]. Distinct miRNA signatures associated with GERD and BE were first described in 2008, and subsequent studies have expanded the list of miRNAs dysregulated in BE to at least 105 miRNAs potentially associated with BE pathophysiology [19, 20, 22–24, 27–32].

This review summarizes the current literature on miRNAs differentially expressed in BE within the context of the major signaling pathways they regulate as a means of highlighting the functional contribution of miRNAs to the onset and progression of BE. miRNAs shown to be dysregulated in BE relative to normal esophageal tissues are listed as upregulated or downregulated in Tables 1 and 2, respectively. Thirteen miRNAs have been identified from serum samples with the potential to serve as circulating biomarkers of BE. Twenty-four of the 105 miRNAs listed were identified as dysregulated in BE in more than one study and validated using multiple methodologies. Of the remaining 81 miRNAs, 53 were shown to be associated with BE in a single study only. Of note, miR-127-3p and miR-200a were reported as both upregulated and downregulated in BE and are thus listed in both Tables 1 and 2. The discrepancy may be due to differences in biopsy content (e.g. goblet cell levels), microenvironment (e.g. associated GERD) or lesion proximity to the squamocolumnar junction for the tissue samples evaluated in these studies. Additional testing is thus needed to clarify the role played by miR-127-3p and miR-200a in BE.

**Table 1:** MicroRNAs downregulated in Barrett's esophagus

MicroRNA	Source^a^	Validated human targets^b^	Method of Detection^c^	References
1-3p	P	NCL, SERP1	9	[27]
10a-5p	P	HOXA1, HOXD10, KLF4, NF1, USF2	3	[27]
106a	T	APP, ARID4B, HIPK3, MYLIP, PAK7, RB1, RUNX1	3,6	[29]
125b	T	ADAMTS1, ALOX5, ANAPC16, ATP6AP1L, B3GALT4, BAK1, BMF, BMPR1B, CASP6, CASP7, CBLN2, CBX7, CDC25A, CDK6, CDKN2A, CEBPG, CYP1A1, DICER1, DIO3, E2F3, ELAVL1, SMO, ST18, ERBB2, ERBB3, KRT19, FAM19A1, GPR160, H3F3A/H3F3B, HIST1H4A, ID1, ID2, ID3, IGFBP3, IL1RN, JARID2, JUN, LIN28A MAN1A1, PCDHB10, PERP, PIGR, RBM8A, SGPL1, TENM2, TRNAK-UUU, TSPAN8, UBE2I, UGT2B15, UGT2B17, UGT2B28	10	[30]
125b-2-3p	T	--	1	[23]
127-3p	P	BAG5, BCL6, COA1, GLE1, PDIA6, PRDM1, SKI, XBP1	9	[27]
1260b	T	BCL6, PRDM1, XBP1	3,5	[113]
133a-3p	P	FSCN1, KLF15, KRT7, PKM, RHOA	9	[27]
133b	T/P	KLF15, PKM, PITX3, PTBP2, STK3	3,7	[20]
136-5p	P	--	9	[27]
149	T	GIT1	4-Feb	[22, 24, 31]
149-5p	T	RAP1A, RAP1B, VAV2	3,5	[113]
154-5p	P	E2F5	9	[27]
18a-3p	T	KRAS	3,6	[29]
193b	T	CCND1, ESR1, ETS1, PLAU	3	[22]
200a	T	BAP1, CTBP2, CTNNB1 (B-catenin), CYP1B1, ELMO2, ERBB2IP, KLHL20, PTPRD, TUBB, WDR37, ZEB1, ZEB2, ZFPM2	3,7	[20]
203	T	ATM, BIRC5, BMI1, LIFR, RAB22A, RUNX2, SCOS1, SIK1, ZEB2	2,3,6-8	[18, 20, 23, 24, 29, 31, 32, 113]
205	T	(-3p): none, (-5p): see below	1-4,6,7	[18, 23, 24, 29, 31, 32]
205-5p	T	ERBB3, INPPL1, MED1, PRKCE, VEGFA, ZEB1, ZEB2	3,5	[113]
20b	T	ARID4B, BAMBI, CRIM1, ESR1, HIPK3, MYLIP, PPARG	3,6	[18]
210	T	FOXN3, SETD2	1,4	[23, 24]
224-5p	T	--	3,5	[113]
23b	T	MET, PLAU	1,4	[23, 24]
27b	T	CYP1B1, MMP13, PPARG, ST14, WEE1	3,4	[22, 24]
3065-5p	T	--	3,5	[113]
31	T	DKK1, PRKCE, TIAM1	3,7	[20]
32	T	BTG2, BCL2L11, MDM2, TSC1	3,6	[18]
33a-3p	T	PBX3	1	[23]
378	T	SUFU	1,4	[23, 24]
378c	T	--	4	[24]
382-5p	P	MXD1	3	[27]
4462	T	--	4	[24]
543	T	NCL	3,6	[18]
708-5p	T	NNAT	3,5	[113]
744	T	BCL2, cMYC, GSK3β, PDCD4, PTEN, PTP1B, SFRP1, TLE3	4	[24]
944	T	PTP4A1, SIAH1	1,2,3	[23, 31, 113]
99a	T	(-5p): FGFR3, IGF1R, MTOR, RPTOR	4	[24]
99a-3p	T	--	1	[23]
Let-7c	T	ACVR1C, APC, APC2, BCL2L1, BMPR1A, BTG2, EIF3J, HMGA2, ITGB3, MED28, mir-30, MYC, PBX2, PPP1R12B, RTCA, SMAD2, SMAD4, STARD13, TGFBR1, TRIB1, TRIM71	1,2,3,6	[23]

^a^ Sample source: tissue (T) or plasma (P).

^b^ Validated human binding targets were obtained from Ingenuity Pathway Analysis Knowledge Database (IPA®, QIAGEN Redwood City, www.qiagen.com/ingenuity).

^c^ Evidence (Table 1): 1: TaqMan Human miRNA Card Set v3.0, 2: Illumina microarray, 3: RT-PCR, 4: Affymetrix GeneChip miRNA 3.0 array, 5: SOLiD 3 sequencing, 6: OSU custom miRNA microarray chip (OSU_CCC version 4.0), 7: Agilent Human Microarray V2; 8: two-color competitive hybridization microarray, 9: mirVana miRNA Bioarrays (Ambion), 10: Exiqon Serum/Plasma Focus miRNA PCR panel.

Underlines indicate validated targets identified in published literature, but not yet listed in the IPA knowledge base.

**Table 2:** MicroRNAs upregulated in Barrett's esophagus

MicroRNA	Source^a^	Validated human targets^b^	Method of Detection^c^	References
122-5p	P	AACS, ADAM17, AKT3, ALDOA, ANK2, ANXA11, AP3M2, ATP11A, ATP1A2, BACH2, BCL2L2, CCNG1, CLDN18, CS, DSTYK, DUSP2, EGLN3, ENTPD4, FAM117B, FOXJ3, FOXP1, FUNDC2, G6PC3, GALNT10, GYS1, MAPK11, MECP2, MEP1A, NCAM1, NFATC1, NFATC2IP, NUMBL, OSMR, PALM, RAB11FIP1, RAB6B, RABIF, SLC7A1, SLC7A11, TBX19, TPD52L2, TRIB1, TTYH3, UBAP2, XPO6	9	[27]
127-3p	T	BAG5, BCL6, COA1, GLE1, PDIA6, PRDM1, SKI, XBP1	3	[114]
130b	T	CYP2C9, CYLD, DICER1, FMR1, IGF-1, MMP2, NKD2, NRP1, PPARγ, PTEN, RUNX3, STAT3	2,3	[31]
133a	T	FSCN1	1	[23]
135b-3p	T	--	3	[114]
136-3p	T	ADAM9, LHR, SLC7A5	1	[23, 114]
143	T	DNMT3A, FNDC3B, KRAS, MAPK7, PLK1, PRC1, TOP2A	1,3,8	[23, 32]
145	T	FOXO1, FOXO3, MYC, SRF	1,3,4,3	[23, 24, 113]
145-3p	T	MTDH, UHRF1	1	[23]
146a	T	ATOH8, BLMH, BRCA1, CCL8, CCNA2, CDKN3, CFH, COL13A1, CXCR4, FADD, KIF22, IRF5, LTB, MCM10, MCPH1, METTL7A, MR1, NFIX, NUMB, PDIK1L, PA2G4, PEX11G, PLEKHA4, PBLD, POLE2, PRR15, RAD54L, SDCBP2, STAT1, TIMELESS, TLR4, TMSB15A, TRIM14, UHRF1, VWCE	2,3	[31]
148a	T	DNMT3B, DNMT1, NR1I2, RPS6KA5	1,4	[23, 24]
151-3p	T	ATP2a2, TWIST1	4	[24]
151-5p	T	SMARCA5, TWIST1	4	[24]
153	T	BCL2, FOXO1, MCL1	2,3	[31]
15a	T	BCL2, CHEK1, WEE1	1	[23]
181a	T	IRF8, MCL1, PBX3	4	[24]
181b	T	AICDA, BCL2, CDX2, CYLD, ESR1, GATA6, GRIA2, NLK, PLAG1, TCL1A, VSNL1	4	[24]
187-3p	T	--	3	[113]
191	T	PTEN, ZEB2	4	[24]
192	T	(-3p): SCD, ALDH3A2	2,3,4,6,7	[18–20, 24, 29, 31]
192-5p	T	DHFR, DTL, TYMS	2,3,5	[31, 113]
194	T	SUFU, (-3p): AP-1, FOSL1	2,3,4,7	[19, 20, 24, 31]
194-5p	S/T	AKT2	2,3,5,9	[27, 31, 113]
195	T	DHFR, DTL, TYMS	1,4	[23, 24]
196a	T	(-3p): LSP1, NRP2, TYMS, ZG16	2,3,4	[24, 31] [19]
196a-5p	T	ANXA1, COL1A1, HOXA7, HOXB8, HOXD8, HOXC8, IKBKB, KRT5, S100A9, SPRR2C	3,5	[113]
196b	T	BCL2, cMYC, GATA6, HOXA9, IGF2BP1, TGFBR2	2,3,4	[31] [24]
1974	S	--	9	[27]
199a-5p	T	ALOX5AP	1	[23, 114]
200a	T	BAP1, CTBP2, CTNNB1, CYP1B1, ELMO2, ERBB2IP, KLHL20, PTPRD, TUBB, WDR37, ZEB1, ZEB2, ZFPM2	2,3	[31]
200b	T	BAP1, CTBP2, CTNNB1, CYP1B1, ELMO2, ERBB2IP, KLHL20, PTPRD, TUBB, WDR37, ZEB1, ZEB2, ZFPM2	2,3,4	[24, 31]
200c	T	ERRFI1, FHOD1, MARCKS, NOG, PLCG1, PPM1F, PTPN13, ZEB1, JAG1	10	[30]
21	T	ACTA2, APAF1, BMPR2, BTG2, C8orf44-SGK3/SGK3, CDC25A, CDK6, CDKN1A, CFL2, CLU, FAM3C, FAS, FBXO11, GLCCI1, HIPK3, IL6R, IRAK1, JAG1, LRRFIP1, MARCKS, MTAP, MYD88, NFIB, PDCD4, PDCD4, PIK3R1, PRRG4, PTEN, RECK, RP2, SERPINB5, SESN1, SLC16A10, SOCS5, SOD3, SOX5, TGFBR2, TIAM1, TIMP3, TNF, TPM1	1,2,3,4	[19, 22–24, 31]
214	T	ING4, PTGS2 (COX2), PTEN	4	[24], -5p [114]
215	T	DHFR, DTL, TYMS	1,2,3,6,8	[18, 23, 29, 31, 32]
223	T	AR, CYB5A, FBXW7, GFPT1, HMGCS1, IRS1, KIF1BP, LMO2, MT1E, NFIA, PARP1, RHOB, SCARB1, SLC11A2, SLC39A1, SMARCD1, SP3, STMN1, STMN1	1	[23], -5p [114]
223-5p	T	IL-6, STAT-3, TP63	1	[23]
25	T	MDM2, TSC1, ZNF512B	1,3,4,5	[23, 24, 113]
26a-1-3p	T	--	1	[23]
28-5p	T	GPX2, IGF1, MAD2, RAP1B, SEPSH2	4	[24]
29c	T	--	1	[23]
29c-3p	T	CDC42, COL1A1, COL1A2, COL3A1, COL4A1, COL4A2, COL15A1, DNMT3A, DNMT3B, FBN1, LAMC1, PIK3R1, SPARC, SRSF10, TDG	1	[23]
301b	T	BIM, NDRG2, TP63	1	[23]
30a-3p	T	CDK6, CYR61, FMR1, SLC7A6, THBS1, TMEM2, TUBA1A, VEZT, WDR82	1	[23]
338-3p	T	UBE2Q1	1,3,5	[23, 113]
3613-5p	T	--	2,3	[31]
375	T	KIAA1524, YAP1, YWHAZ	4	[24]
376c-3p	T	--	3	[114]
409-3p	T	AKT1, ANG, Beclin-1, cMET, CTNND1, GAB1, PHF10, ZEB1	1	[23, 114]
424-5p	T	ANLN, ATF6, CCND1, CCNE1, CCNF, CDC14A, CDC25A, CHEK1, FGF2, FGFR1, KIF23, GALNT13, MAP2K1, mir-9, MGAT4A, NFIA, OGT, PLAG1, SPI1, WEE1	1,2,3	[23, 31, 114]
4417	T	--	4	[24]
450b-5p	T	MET, SOX2	1	[23]
451a	S	ABCB1, AKTIP, FBXO33, MIF	9	[27]
487b	T	CDKN2AIP, MAP2K4	3	[22]
492	T	CD147, PTEN, RETN, SOX7	1	[23]
497	T	BCL2, BDNF, E2F3, FRA1, HIF1a, HDGF, KCa3.1, mTOR, NRDP1, SIRT4, SLUG, SMAD7, VEGFA, VEGFR2, YAP1	1	[23]
501-5p	T	CYCLD, DKK1, GSK3β, NKD1	1	[23]
503	T	AGO1, ANLN, ATF6, CCND1, CCNE1, CCNE2, CCNF, CDC14A, CDC25A, CDKN1A, CHEK1, FGF2, FGFR1, mir-9, WEE1	4	[24]
542-3p	T	ILK, PTGS2 (COX2)	1	[23]
548b-3p	T	--	3,6	[18]
551b-3p	T	--	3,5	[113]
618	T	XIAP	1	[23]
625	T	(-3p): MAP2K6, SCAI; (-5p): NTRK3	2,3	[31]
642	T	--	1	[23]
7	T	EGFR, IRS1, IRS2, KMT5A, NEFM, PAK1, RAF1, SLC3A2, SNCA	2,3	[31]
95-3p	S	CELF2, CCND1	9	[27]
Let-7	T	MYC	4	[24]

^a^ Sample source: tissue (T), plasma (P) or serum (S).

^b^ Validated human binding targets were obtained from Ingenuity Pathway Analysis Knowledge Database (IPA®, QIAGEN Redwood City, www.qiagen.com/ingenuity).

^c^ Evidence (Table 2): 1: TaqMan Human miRNA Card Set v3.0, 2: Illumina microarray, 3: RT-PCR, 4: Affymetrix GeneChip miRNA 3.0 array, 5: SOLiD 3 sequencing, 6: OSU custom miRNA microarray chip (OSU_CCC version 4.0), 7: Agilent Human Microarray V2, 8: two-color competitive hybridization microarray; 9: Exiqon Serum/Plasma Focus miRNA PCR panel, 10: mirVana miRNA Bioarrays (Ambion).

Underlines indicate validated targets identified in published literature, but not yet listed in the IPA knowledge base.

Validated human target genes for each of the BE-associated miRNA are also listed in Tables 1 and 2. The target genes listed were obtained from the Ingenuity Pathway Analysis Knowledge Database (IPA®, QIAGEN Redwood City, www.qiagen.com/ingenuity), a curated database of findings from peer-reviewed scientific publications. The genes listed are high confidence interactions based on validation in human tumor cell lines. This list was chosen as a conservative list of functional targets for discussion herein, as it is impossible to discuss the full list of 10,512 predicted targets identified using Partek Genomics Suite (PGS, version 7.17, Target Scan 7.0 database query). In addition to the target genes listed in the IPA database, several additional targets have been included based on reports in the literature. These targets are underlined in Tables 1 and 2. Although the literature on signaling pathways in BE and EAC is extensive and spans over six decades, this review only focuses on miRNAs that are differentially regulated in BE and regulate Hedgehog, Notch, MAPK, NFκB, Wnt, and EMT signaling thought to underlie BE onset and progression.

## THE SQUAMOUS TO COLUMNAR TRANSITION OF BE: MIRNAS IN HEDGEHOG AND BMP SIGNALING

**Figure 1:**
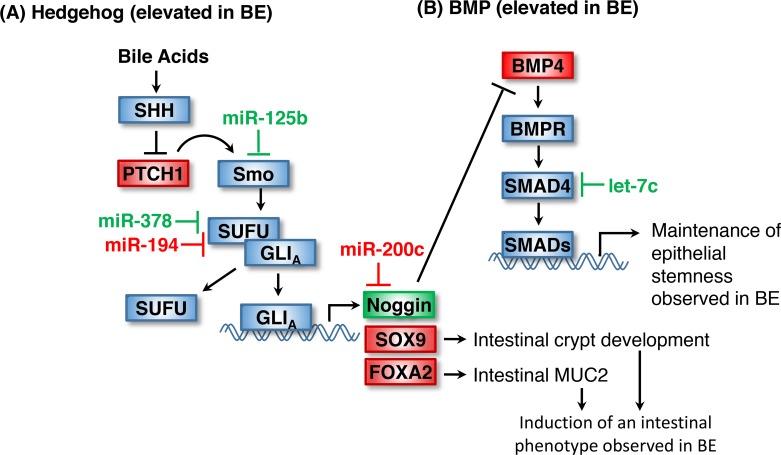
Maintenance of stemness in BE by Hedgehog and BMP signaling **(A)** Upregulation of Hedgehog signaling in BE is thought to occur as an adaptive response to chronic bile acid exposure in GERD. Loss of miR-125b and increased expression of miR-194 in BE would de-repress Smoothened and SUFU respectively and serve to facilitate SHH signaling. **(B)** Increased BMP signaling in BE is thought to result from a loss of the HH target Noggin, potentially a result of elevated miR-200c expression. Loss of Let-7c may also contribute to increased BMP signaling. Items shown in red and green are upregulated and downregulated in BE, respectively.

BE is characterized by the conversion of normal squamous epithelium to an intestinalized columnar epithelium resembling an embryonic architecture. The molecular basis for this conversion is under debate, but it's believed to arise from a residual esophageal stem cell population as an adaptive response to chronic gastric reflux [33, 34]. Hedgehog (HH) signaling is thought to play a role because it is critical for regulating adult stem and progenitor cells during homeostasis and disease. HH signaling is absent in the normal adult esophagus but frequently upregulated in Barrett's epithelium, and 96% of EAC patients have elevated HH target gene expression [35, 36]. HH signaling is stimulated by the ligands Desert/Indian/Sonic Hedgehog (D/I/SHH) binding the Patched-1 (PTCH1) receptor. Ligand binding to PTCH1 relieves inhibition of Smoothened (SMO), a G-protein coupled receptor-like protein, resulting in cleavage of the SHH repressor, Suppressor of Fused (SUFU) from the GLI transcription factors (Figure 1A). This cleavage allows the GLI proteins to initiate transcription of HH target genes [37].

miRNA regulation of SHH signaling in BE potentially occurs through miR-125b, which targets smoothened (SMO) and is downregulated in BE (Table 1), consistent with the expected increase in Hedgehog-responsiveness in BE [23]. Overexpression of miR-125b has previously been observed in lung carcinoma cell lines, is associated with poor prognosis in HER2-positive breast cancer patients and is dysregulated in neuronal progenitor tumor cells. Although these studies indicate miR-125b has oncogenic potential, its functional role in BE has not been investigated [38–40]. miR-194 targets SUFU and is upregulated in BE tissues [41]. The resulting loss of SUFU would lead to increased GLI transcriptional activity and HH target expression, as observed in BE. miR-378 is also known to target the SHH pathway through its targeting of SUFU, a negative regulator of SHH signaling, but its downregulation in BE (Table 1) is inconsistent with the observed increase in SHH signaling [42]. miR-378 is reduced in EAC, suggesting that it may have diagnostic or prognostic value, but its role in BE physiology remains unclear [23, 24].

Ectopic HH signaling blocks the expression of Noggin, an inhibitor of the BMP (bone morphogenic protein) pathway, leading to increased BMP4 signaling and resulting in failed stratification of the columnar epithelium and a BE phenotype [43]. BMP signaling promotes expression of stem cell genes while suppressing genes involved in cell death. BMP signaling is increased in BE and is thought to function in the maintenance of epithelial stemness in BE [44]. BMP signaling occurs through the receptor-mediated SMADs (SMAD1/5/8) which associate with the co-mediator SMAD4 and are translocated to the nucleus to activate BMP target gene expression [45]. Increased bile acids associated with GERD and BE have been shown to directly stimulate HH signaling and increase expression of PTCH1 and BMP4 in the BE stroma [36]. Other downstream targets of elevated HH in BE include SOX9, a transcription factor important in intestinal crypt development, and FOXA2, a driver of the intestinal mucin MUC2 [46, 47]. Collectively, these studies suggest bile reflux elevates HH signaling which induces an intestinal phenotype through BMP4, SOX9 and FOXA2 in the esophageal squamous epithelium.

The elevated BMP signaling observed in BE is potentially mediated by miR-200c, miR-130 and let-7c (Figure 1B). miR-200c is upregulated in BE (Table 2) and directly targets and inhibits Noggin expression, and thus is a potential contributor to the enhanced BMP signaling observed in BE [30, 48]. Let-7c is downregulated in BE and may contribute to increased BMP signaling by increasing both SMAD2 and SMAD4 levels [23, 49].

## THE SQUAMOUS TO COLUMNAR TRANSITION OF BE: MIRNAS AND NOTCH SIGNALING

**Figure 2:**
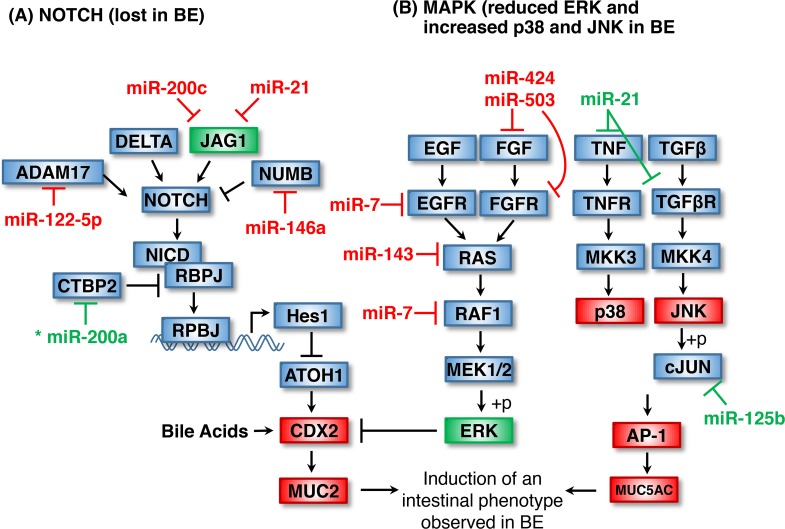
Loss of epithelial stratification and intestinalization of the columnar epithelium by Notch, CDX2 MAPK signaling **(A)** Loss of NOTCH signaling in BE is thought to occur as a consequence of diminished JAG1 ligand expression which may result from an upregulation of miR-200c and miR-21 in BE. Enhanced miR-122-5p expression may abrogate NOTCH signaling through repression of the NOTCH agonist ADAM17. Loss of miR-200a potentially contributes to NOTCH suppression by alleviating repression of the co-repressor CTBP2 and leading to destruction of the NICD/RBPJ transcriptional complex. Loss of NOTCH signaling reduces HES1 expression, relieving ATOH1 inhibition and leading to increased CDX-2 and MUC2 levels observed in BE. **(B)** Loss of ERK signaling in BE may be explained by an upregulation of several BE-associated miRNA, including miR-424, miR-503, mir-7, miR-143 and miR-7. Loss of ERK has also been linked to increased CDX2 levels. Loss of miR-21 and miR-125b may contribute to increased p38 and JNK kinase levels observed in BE. Asterisk indicates miRNA with unclear regulation in BE. Items shown in red and green are upregulated and downregulated in BE, respectively.

In the normal esophagus, Notch signaling is strongest in the basal layer of stratified epithelia, where it maintains normal differentiation and stratification [50]. Signaling is induced when Delta-like ligand 1/2 (DLL1/2) or Jagged1/2 (JAG1/2) interacts with the NOTCH1/2 receptor, resulting in cleavage of its intracellular domain (NICD) (Figure 2A). NICD combines with recombination signal binding protein for immunoglobulin kappa J region (RBPJ) and Mastermind-like proteins (MAML), forming a transcriptional complex [51]. JAG1 is frequently downregulated in BE resulting in the loss of Notch signaling and epithelial stratification [52]. Interestingly, Notch signaling is elevated during the progression of BE to EAC suggesting that miRNA regulation of Notch signaling may be disease stage-dependent [53].

As shown in Table 2, miR-21, miR-200c, miR-122-5p and miR-146a are upregulated in BE and target the Notch pathway (Figure 2A) [24, 27, 30]. miR-21 and miR-200c target JAG1 resulting in loss of NOTCH activation, consistent with a role in mediating the squamous to columnar transition in BE [54, 55]. miR-122-5p is also upregulated in BE and targets the Notch agonist ADAM17, potentially reducing the ADAM17-mediated cleavage of NOTCH and downstream signaling [27, 56, 57]. MiR-146a is upregulated in BE and has been shown to target the Notch antagonist NUMB, raising the possibility that miR-146a is involved in re-establishing Notch signaling upon dysplastic progression of BE [53, 58]. Finally, miR-200a inhibits the C-terminal binding protein 2 (CTBP2) which serves as a co-repressor of RBPJ [59]. Loss of miR-200a would lead to increased CTBP2 levels and could account for the inhibition of Notch signaling expression in BE, but there are conflicting reports on how miR-200a expression is altered in BE.

## INTESTINALIZATION OF THE COLUMNAR EPITHELIUM: MIRNAS IN CDX2 AND MAPK SIGNALING

The Caudal Type Homeobox 2 (CDX2) transcription factor drives the expression of intestinal genes and those involved in goblet cell differentiation and is upregulated in BE (Figure 2) [60]. Increased CDX2 levels in BE are promoted by reduced Notch signaling, unconjugated bile acids in GERD, activation of p38, and by the inactivation of ERK signaling [17, 61–63]. Although increased activation of the ERK Mitogen Associated Protein Kinase (MAPK) has been observed in GERD patients without BE, reduced phosphorylation of the ERK is frequently observed in Barrett's epithelium relative to the normal squamous epithelium suggesting that ERK activation by gastric acid is abrogated in BE [61, 64, 65]. ERK inactivation in BE is thought to promote the observed increase in CDX2 transcriptional activity and promote the terminal differentiation of intestinal epithelial cells [66].

As shown in Table 2, several miRNAs upregulated in BE are predicted to target the ERK pathway (Figure 2B). ERK is activated by the binding of growth factors (EGF, FGF) to their receptors (EGFR, FGFR). EGFR is targeted by miR-7, while miR-424 and miR-503 target both FGF and the FGFR [67, 68]. Reduced ERK activation occurring as a consequence of EGFR and FGFR inhibition by these miRNAs potentially explains the observed reduction in signaling through the RAF-RAS-MEK-ERK kinase cascade in BE [69]. Within the pathway itself, upregulation of miR-143 and miR-7 observed in BE may also contribute to the loss of ERK signaling through downregulation of KRAS and RAF1, respectively [70, 71].

Of note, increased MAPK signaling through JNK and p38 is believed to contribute to the intestinalization in BE through induction of activated AP-1, a transcription complex that includes JUN and FOS heterodimers [72]. AP-1 is responsible for the expression of MUC5AC in BE lesions, as well as several genes involved in inflammation and carcinogenesis [73–75]. miR-21 is known to inhibit the TNF ligand and the TGFβ receptor, upstream activators of p38 and JNK, respectively. miR-21 is downregulated in BE, consistent with an increase in both signaling cascades and consistent with a role for miR-21 in BE (Table 1) [24]. miR-125b targets cJUN and is also downregulated in BE, thus potentially leading to an increase in other factors downstream of JNK (Table 1) [30, 76].

## DYSPLASTIC PROGRESSION TO EAC: MIRNAS IN WNT SIGNALING

**Figure 3:**
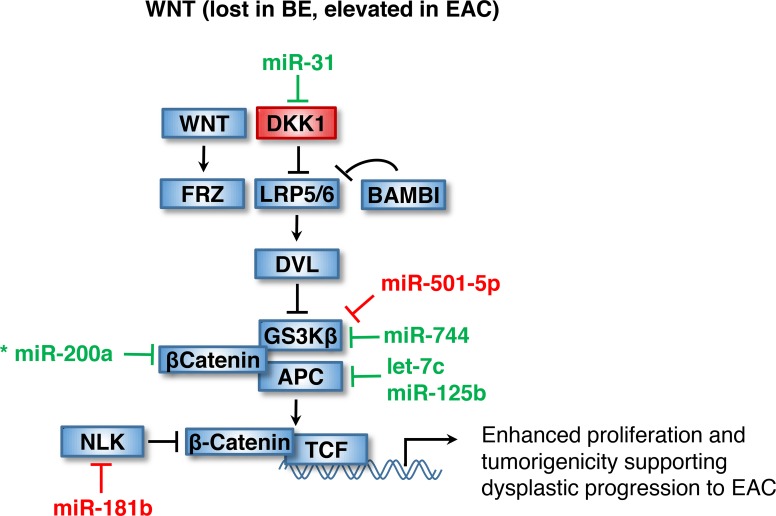
Dysplastic progression to EAC through WNT activation WNT signaling is lost in BE but elevated during dysplastic progression to EAC. Loss of WNT signaling in BE is thought to occur as a consequence of elevated DKK1 levels, potentially resulting from miR-31 loss. Subsequently, loss of let-7c, miR-125b and miR-200a and increased miR-181b levels may contribute to WNT activation in EAC by facilitating accumulation of nuclear β-catenin and activation of WNT target genes. Asterisk indicates miRNA with unclear regulation in BE. Items shown in red and green are upregulated and downregulated in BE, respectively.

Wnt signaling is critical for normal intestinal development and homeostasis (Figure 3), and is thought to play a critical role in the dysplastic progression of BE to EAC [77]. Wnt signaling is normally active in the esophagus after its separation from the foregut, but is absent in BE due, in part, to an upregulation of the WNT inhibitor Dickkopf-related protein 1 (DKK1) [78, 79]. Increased DKK1 prevents recruitment of glycogen synthase kinase-3β (GSK3β) and *Adenomatosis Polyposis Coli* (APC) to the cell membrane, thus facilitating β-catenin phosphorylation and degradation. The resulting loss of nuclear β-catenin reduces transcription of Wnt target genes as observed in BE [80]. By contrast, increased nuclear β-catenin is observed in high-grade dysplasia suggesting that canonical WNT/β-catenin activation does not contribute to the onset of BE but instead promotes the dysplastic progression of BE to EAC [78, 81]. SOX17, a negative regulator of WNT, is progressively lost in the progression from normal mucosa to EAC, but it remains unclear whether SOX17 directly contributes to the metastatic progression to EAC [82]. miR-141 has been shown to downregulate SOX17 expression in esophageal cancer cell lines, thus potentially activating Wnt signaling to promote esophageal tumorigenesis, but miR-141 dysregulation in BE or EAC has not yet been reported [82].

Loss of WNT activation in BE is potentially linked to a downregulation of miR-31, miR-744, let-7c and miR-200a in BE, although the contribution of these miRNAs to the progression to EAC remains unclear (Table 1). miR-31 directly targets the Wnt inhibitor DKK1, thus providing a possible explanation for the observed increase in DKK1 levels in BE [20, 83]. The subsequent elevation of miR-31 in EAC relative to BE suggests a potential role for miR-31 in the progression to EAC in a manner similar to its role in lung cancer where it is known to enhance proliferation and tumorigenicity through upregulation of Wnt signaling [20, 84]. miR-744 has also been shown to directly target several negative modulators of Wnt/β-catenin signaling, including glycogen synthase kinase 3β (GSK3β) [15]. The observed loss of miR-744 is thus consistent with diminished Wnt signaling in BE, but unlike its role in driving WNT activation and tumorigenicity through increased expression in pancreatic cancer, miR-744 is instead reduced in EAC calling its role in dysplastic progression to EAC into question [85]. Loss of let-7c potentially leads to an increase in APC levels and stabilization of the destruction complex and may serve to limit β-catenin transactivation in BE [24, 38, 49]. Like miR-744, Let-7c is subsequently reduced in EAC [21].

A number of BE-related miRNAs have targets in the Wnt signaling cascade and potentially play a role in promoting Wnt signaling during its metastatic progression toward EAC. Upregulation of miR-181b in BE decreases Nemo Like Kinase (NLK), alleviating repression of TCF and facilitating TCF/LEF binding to Wnt-response elements and increasing Wnt target gene expression (Table 2) [24, 86]. Although a definitive link to BE or EAC has not been established, miR-181b, shown to increase tumor metastasis in a mouse model of non-small cell lung carcinoma (NSCLC), is upregulated in both metastatic human NSCLC and breast cancer and is regulated by CDX2 [87, 88]. miR-501-5p may similarly activate Wnt signaling by inhibiting GSK3β, thus promoting malignant behavior in EAC as previously observed in gastric cancer [89]. Additionally, loss of miR-200a in meningiomas was shown to promote tumor growth by directly targeting beta-catenin (CTNNB1), but its role in BE remains unclear due to conflicting reports on the dysregulation of miR-200a [20, 31, 90].

Several other miRNAs differentially regulated in BE appear to regulate cell cycle progression through modulation of downstream validated Wnt targets (Tables 1 and 2). miR-145 and let-7 target c-MYC, miR-125b targets c-Jun, and miR-424 and miR-503 target cyclinD1 [76, 91–94]. These miRNAs appear to have contrasting functions, thus further studies into their roles in mediating BE-physiology are needed.

## ELEVATED NF-ΚB SIGNALING AND EMT IN EAC

**Figure 4:**
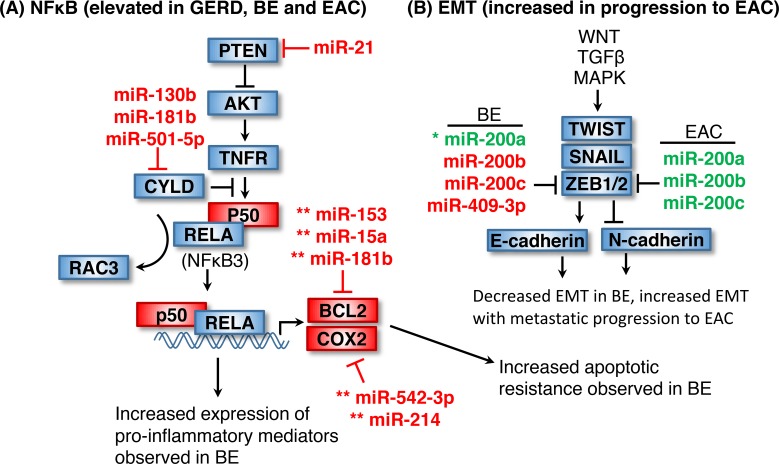
Elevated NF-κB signaling and EMT in EAC **(A)** NF-κB signaling is elevated in GERD, BE and EAC, potentially due to increased miR-130b, miR-181b or miR-501-5p-mediated suppression of the NF-κB suppressor CYLD. BCL2 and COX2 are elevated in BE as a result in increased NF-κB signaling. The miRNA which target these mRNA (marked with an asterisk) are upregulated and inconsistent with a role in BE or EAC. **(B)** EMT is increased during the MDC progression to EAC. The miR-200 family of miRNA and miR-409-3p are increased in BE and thus potentially function to suppress ZEB1 and ZEB2 during early stages. miR-200 family miRNAs are subsequently suppressed in EAC, thus correlating with increased ZEB1 and ZEB2 and thus EMT. Asterisk indicates miRNA with unclear regulation in BE. Double asterisk indicates miRNA regulation inconsistent with a role in BE or EAC. Items shown in red and green are upregulated and downregulated in BE, respectively.

Although not normally expressed in squamous esophageal epithelium, NF-κB signaling is elevated in a variety of cancers, inflammatory diseases, and is increased in GERD, BE, and EAC tissue relative to adjacent normal squamous epithelium [95]. NF-κB is elevated in 40% of BE patients, and its expression correlates strongly with dysplastic progression to EAC [96, 97]. Unconjugated bile acids induce NF-κB signaling, increasing the expression of a variety of downstream pro-inflammatory mediators frequently overexpressed in BE and esophageal cancer cell lines [96, 98, 99]. miR-21, miR-130b and miR-181b are upregulated in BE and are known to positively regulate NF-κB signaling (Figure 4A). miR-21 targets PTEN, which results in an increase in AKT signaling and downstream NF-κB [100]. miR-130b, miR-181b and miR-501-5p are known to drive inflammation through NF-κB by targeting the Ubiquitin-Specific-Processing Protease (CYLD), a known NF-κB suppressor [100–102]. Mechanistic studies into the role(s) played by these miRNAs in regulating NF-κB signaling in BE and EAC are needed.

B-Cell CLL/Lymphoma 2 (BCL2) and Cytochrome C Oxidase II (COX2), both downstream NF-κB effectors, are upregulated and associated with apoptotic resistance in BE [103, 104]. Several BE-related miRNAs which target these genes are upregulated (indicated in Figure 4A with a double asterisk). Upregulation of these miRNAs (shown in Figure 4A) would suppress NF-κB signaling and is not consistent with the observed increase in NF-κB signaling BE or EAC. However, the corresponding miRNA mimics may hold therapeutic potential by increasing apoptosis in BE lesions.

In the transition from BE to invasive EAC, the Epithelial-Mesenchymal transition (EMT) is a prerequisite for tumor invasion (Figure 4B). EMT, a process by which epithelial cells become migratory and invasive, is regulated by several transcription factors including ZEB1/2 and SNAIL1/2 [105]. The miR-200 family of miRNAs, including miR-200a, miR-200b and miR-200c are known to regulate EMT through inhibiting ZEB1/2, but a role in the BE to EAC progression has not been clarified. These miRNAs are upregulated in BE and suppressed in EAC, consistent with a role in promoting EMT during the dysplastic progression to EAC [106, 107].

## CONCLUSIONS

While a number of the key regulators underlying BE pathophysiology and its progression to EAC have been identified, effective protein and miRNA biomarkers for BE remain poorly defined. As regulators of a third of all human genes, miRNAs have potential beyond clinical biomarkers and may be used to elucidate the interactions among signaling pathways in BE pathogenesis, as well as other human diseases. This review strived to provide mechanistic context by correlating the key regulatory proteins involved in BE with miRNAs that are differentially expressed in BE. Using IPA, we were able to correlate miRNAs dysregulated in BE to the intestinalization of esophageal tissue through upregulation of Hedgehog signaling, and in the maintenance of epithelial stemness in BE through upregulation of BMP4 signaling. We further elaborated on the potential mechanisms by which dysregulated miRNAs downregulate NOTCH and ERK signaling while upregulating JNK and p38 signaling to induce mucin expression and maintain intestinal identity. We identified miRNAs which potentially play a role in regulating proliferation and tumorigenicity by suppressing WNT signaling in BE and enhancing WNT signaling during the dysplastic progression to EAC. We illustrate how miRNAs which are dysregulated in GERD, BE and EAC support increased NFkB signaling and expression of pro-inflammatory mediators and apoptotic resistance. Finally, identification of several miRNAs which affect EMT signaling were correlated to the metastatic progression to EAC.

Few studies have compared miRNA signatures from tissue and serum, so it is difficult to predict whether circulating miRNA levels accurately reflect those from esophageal biopsies [108]. Of the 105 BE-related miRNAs discussed in this review, only 13 were characterized from serum or plasma. Additional testing will be required to determine if the remaining miRNAs tested from tissue are part of the circulating miRNA signature of BE. Strong correlations between serum and tissue miRNA levels observed in breast and non-small cell lung cancer signatures is promising, but additional studies are needed in order to determine if BE-related miRNAs identified from tissue samples (Tables 1 and 2) serve as effective circulating biomarkers [109, 110]. Bansal et al. utilized muscle biopsies from BE patients with or without dysplasia to identify dysregulation of miR-15b, miR-21, miR-203, miR-485-5p and let-7a as collectively predictive for dysplasia in BE [26]. Determining whether expression of these miRNAs is similarly altered in serum would provide an initial gauge of how effectively serum miRNA profiles reflect those in BE lesions.

Transduction of human cancer cells with lentiviral vectors encoding for antisense oligos to selected miRNAs has been shown to reduce pancreatic tumor burden in SCID mice [111]. Accordingly, it is our hope that knockdown of key miRNAs overexpressed in BE (Table 2) may hold therapeutic potential. In particular, knockdown of miR-122-5p, miR-21, miR-200c, miR-7, miR-143, miR-503, mir-424 and miR-181b are of particular interest due to their functional ties to key regulators of BE progression in the NOTCH, MAPK, EMT, Wnt, NFκB and EMT pathways (Figures 1–4).

While this review focused on the downstream targets of miRNAs known to be dysregulated in BE, it should also be noted that these same miRNAs may be used to predict potential upstream master regulators which mediate BE lesion formation. At least 12 of the 98 BE-related miRNAs are direct targets of p53, suggesting that p53 may be causal in some fashion. Specifically, miR-143, miR-145, miR-191, miR-192, miR-22, miR-25, miR-661 and let-7 are upregulated in BE and validated targets of p53. miR-149, miR-210, miR-32 and miR-378 are downregulated by p53. A role for p53 in BE has not been clearly defined, but elevated p53 expression (>5%) is associated with progression to advanced neoplasia [112].

Identification of clinically reliable early miRNA biomarkers of BE will require extensive validation and a deeper understanding of the cellular signaling events which drive BE development. Of the miRNAs discussed in this review, it remains unclear which miRNAs have the potential to serve as biomarkers specific to BE and which are broad spectrum cancer biomarkers. Further studies into the mechanisms by which circulating miRNAs become differentially expressed are needed to identify those miRNAs of real clinical importance. A number of technical challenges remain which have hindered current efforts at identifying miRNA biomarkers including low miRNA yield from serum samples, lack of suitable endogenous miRNA controls, and a lack of strategies to deal with normal variation in circulating miRNA levels. Improved early detection of BE and other cancers will only occur by overcoming these technical challenges and by obtaining a more detailed understanding of miRNA signaling networks.

## Abbreviations

BE: Barrett's esophagus
BMP: bone morphogenic protein
EAC: esophageal adenocarcinoma
EMT: epithelial-to-mesenchymal transition
GERD: gastroesophageal reflux disease
HH: hedgehog
MAPK: map kinase
MDC: metaplasia-dysplasia-carcinoma
miRNA: microRNA
WLE: white light endoscopy.
